# Implementing youth advisory boards with sexual minority adolescents and young men: sharing experiences, challenges and opportunities from East, South, and West Africa

**DOI:** 10.1186/s13063-026-09596-5

**Published:** 2026-03-11

**Authors:** Sylvia Adebajo, Taylor Lascko, Marie-Claude C. Lavoie, Akipu Ehoche, Chester Mutumba, Nicholas Shima Aernan, Nathan Nganga, Benson Njoroge, Philip Daniel Chinseu, Richard Gichuki, Olawole Ayorinde, Ojore Godday Aghedo, Kennedy Sambambi, Gift Ndalumbira, Maria Chinoko Ngulube, John Chama, Elizabeth Shoyemi, Gift Trapence, Joshua Kimani, Cassidy W. Claassen, Nadia A. Sam-Agudu, Lisa Hightow-Weidman, Man Charurat, RISE Study

**Affiliations:** 1 Institute of Human Virology, University of Maryland School of Medicine, Baltimore, MD USA; 2 Institute of Human Virology Nigeria, Abuja, Nigeria; 3 Division of Global Health Sciences, Department of Epidemiology and Public Health, University of Maryland School of Medicine, Baltimore, MD USA; 4 Ciheb Zambia, Lusaka, Zambia; 5 International Centre for Advocacy On Right to Health, Abuja, Nigeria; 6 Partners for Health and Development in Africa, Nairobi, Kenya; 7 Centre for the Development of People, Lilongwe, Malawi; 8 Centre for Population Health Initiatives, Lagos, Nigeria; 9 Global Pediatrics Program and Division of Pediatric Infectious Diseases, University of Minnesota Medical School, Minneapolis, MN USA; 10 Institute On Digital Health and Innovation, College of Nursing, Florida State University, Tallahassee, FL USA

**Keywords:** Youth advisory boards, Adolescents, Young adults, Young sexual minority men, Experiences, Challenges, Opportunities, Clinical trials, Sub-Saharan Africa

## Abstract

Clinical trials involving sexual minority adolescents and young men in low- and middle-income countries have historically been limited due to a combination of structural, social, and scientific barriers that often hinder their participation. In addition, researchers lack the cultural competence or knowledge of inclusive recruitment strategies to effectively engage these populations. In this commentary, we describe the experiences, challenges, and opportunities in establishing youth advisory boards as a pathway to entry into the community, overcoming exclusion, building trust, and incorporating the voices of under-served sexual minority adolescents and young men in clinical trials and the development of community-informed interventions.

**Trial registration:** ClinicalTrials.gov NCT06350682. Registered on February 10, 2026.

## Background

In sub-Saharan Africa (SSA), where same-sex sexual behaviors are often criminalized [1], sexual minority adolescents and young adults (SM-AYA) face higher risk factors but have less access to services. In particular, young (ages 15–24 years) men who have sex with other men (MSM) and transgender women (TGW) are disproportionately burdened with higher prevalence and incidence of HIV [2–5], depression, anxiety, and suicidal ideation [6] compared to their cisgender and heterosexual counterparts. Yet, SM-AYA who are most in need of innovative, evidence-based HIV biomedical and behavioral interventions remain poorly engaged or represented in clinical trials, the findings of which could benefit them [7, 8]. Consequently, clinical trials involving these sub-populations have historically been limited largely due to a combination of structural, and social, barriers. Stigma, discrimination, violence and hostility often discourage participation in research [9], and researchers may also lack the cultural competence or the knowledge of inclusive recruitment strategies to effectively engage them [1, 3]. Another challenge in engaging SM-AYA in clinical trials, specifically those under the age of 18, is the regulatory requirement for parental or guardian consent [8]. This exclusionary requirement poses a hindrance, particularly for SM-AYA who are hesitant to disclose their sexual and gender identities to their families due to fear of rejection [10]. Additionally, the concept of “emancipated or mature minors”, which allows adolescent girls (especially those who are pregnant) to be deemed sufficiently mature to consent independently for clinical trial participation, is not widely recognized or uniformly applied across SSA for adolescent males. These multi-faceted barriers have contributed to gaps in knowledge on how to effectively reach and deliver evidence-based interventions tailored to the unique needs and context of SM-AYA.

Meaningful engagement of SM-AYA in the lifecycle of clinical trials from co-design and co-production to dissemination aimed at developing effective, culturally tailored, evidence-based, community-informed interventions can be achieved through the establishment of community advisory committees [11–13]. Youth Advisory Boards (YABs) are community advisory committees that allow meaningful involvement of youth in the design and implementation of youth research projects. YABs provide a pathway for overcoming exclusion, building trust, and incorporating the voices of SM-AYA in clinical trials to improve HIV treatment and prevention outcomes [14–16]. However, the literature is sparse on tested theoretical frameworks for establishing and maintaining YABs [14, 17]. Youth engagement aligns with the 1989 United Nations’ Convention on the Rights of the Child [18], not only as a fundamental right to be involved in matters concerning them, but it is also a requirement to achieve the health-related Sustainable Development Goals by 2030 [19–21].

**Fig. 1:**
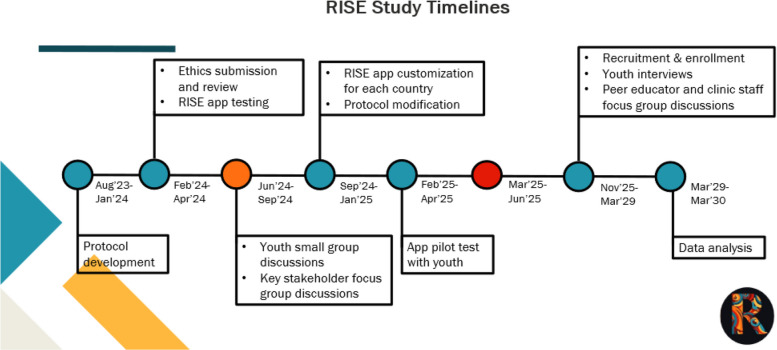
RISE study timelines

This paper describes the experiences, challenges, opportunities and lessons learned in establishing YABs for the Resilient HIV Implementation Science with Young Men Using Evidence (RISE) Clinical Research study, designed to improve HIV prevention and treatment through the use of youth-tailored mobile health application, HealthMpowerment [22–26], across four countries – Kenya, Malawi, Nigeria, and Zambia based on UNICEF’s guidelines on adolescent participation and civic engagement [27]. RISE is a five-year clinical trial (2023 – 2029). Participant enrollment is scheduled for early 2026 having completed multiple pre-trial milestones (Fig. 1).

Lessons learned in establishing the RISE YABs across four countries will benefit other researchers to promote youth inclusion and empowerment, build credibility, and trust with youths, and develop the next generation of youth leaders and advocates in health services and research.

### YAB member selection, recruitment, and roles

To promote community buy-in and foster a strong sense of ownership among adolescents and youth stakeholders, the intended users of the RISE app, all study sites recruited SM-AYA to serve as members of the YAB in each country. These boards are involved throughout the study duration to ensure the priorities and interests of SM-AYA are reflected in the design, implementation, and evaluation of the project. All YAB members were recommended by their peers based on their level of influence, trustworthiness, acceptance within their networks, willingness and confidence in their abilities to represent the interests of their communities. Recruitment to join the YAB was based on their level of involvement as peer leaders in their communities and study sites. In addition, potential YAB members had to meet the following criteria to be recruited: (i) be between the ages of 15 and 24 years (16 and 24 years in Zambia in accordance with national regulations); (ii) reside in the community; (iii) identify as MSM or TGW; and, (iv) report living with HIV or at a high likelihood of exposure to HIV. Members were provided with detailed information about the RISE study, their responsibilities, potential risks and benefits of participation, and safeguard measures. YAB members who reach the age of 25 during the study are asked to step down and are replaced, ensuring the YAB remains representative of the community from which study participants are recruited. The RISE study’s longstanding collaboration with trusted community-based groups, clinics, and SM-AYA–friendly safe spaces has facilitated identification and recruitment of YAB members. Other positive factors include involving community gatekeepers at the outset with detailed information about the study’s goals, YAB expectations, promises of privacy, and outlining the benefits for YAB members.

YAB member roles include acting as liaisons between SM-AYA communities and the RISE study team. Besides being involved in the study planning, implementation, and dissemination of results, YAB members are actively engaged in demand creation for the RISE mobile app, providing inputs on strategies for recruiting, retaining, and re-engaging SM-AYA study participants. Furthermore, YAB meetings are intentionally structured to promote inclusive participation, facilitated discussions, small-group activities, and creativity in identifying multiple avenues for contribution (e.g., written feedback, virtual engagement), ensuring that the less outspoken members are encouraged to meaningfully participate.

### YAB structure

**Fig. 2:**
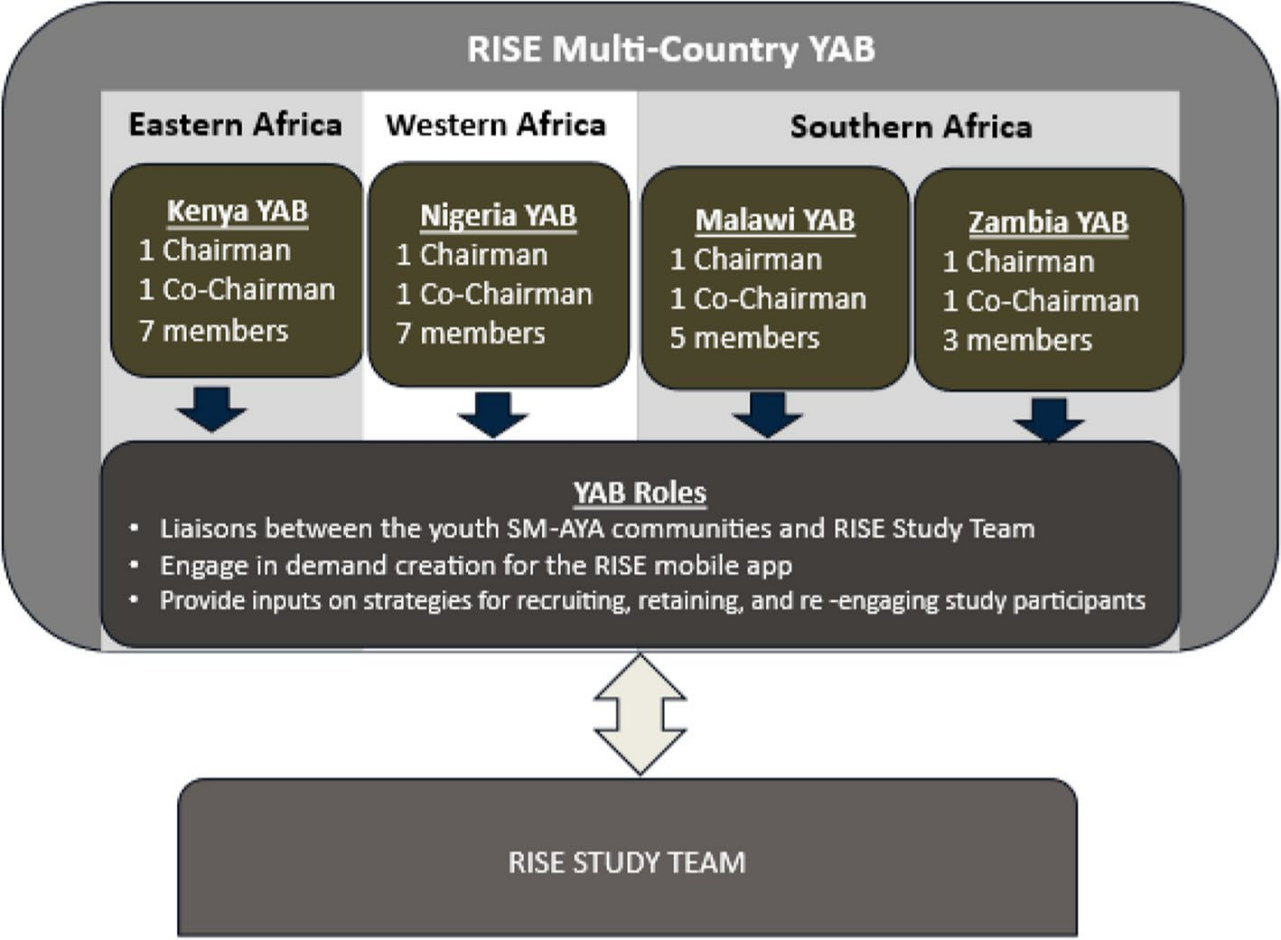
YAB governance, structure, and roles

Across each participating country, a YAB chairman and co-chairman were nominated by peer members to preside over quarterly and any additional YAB meetings, liaise with RISE leadership, and represent their country’s YAB in biannual RISE multi-country YAB meetings (Fig. 2).

While physical meeting attendance is scheduled at least once per year, most meetings take place virtually or in a hybrid style where members who live closer together converge at safe spaces or clinics and join the rest of the group virtually.

In addition, to the roles listed in Fig. 2, YAB members also perform the following functions – represent their peers’ interests in all community activities; actively engage in the planning, implementation and dissemination of the study’s findings; advise the study as it relates to inclusion of community voices in the implementation of their project and actively engage in quarterly virtual and/or physical YAB meetings. To properly guide their operations, these roles were defined in the YAB standard operating procedure and terms of reference.

### Member characteristics

Each country established a YAB with five to nine members (30 in total). The YABs include diverse community representatives, comprising 17 MSM (56.7%) and 13 TGW (43.3%). Thirty-seven percent of the YAB members were 19 years and below, and a third (33.3%) were living with HIV. While a similar proportion of YAB members were employed (30.0%) or students (30.0%), the remaining participants were unemployed (40.0%).

### YAB engagement

Since the inception of the RISE study, YAB members actively participated in adapting and testing the RISE app. During this phase, they identified app technical issues, and recommended improvements, such as educational games, medication reminder notifications, and a comprehensive drug list beyond the HIV medications listed on the app. Furthermore, they ensured that the app’s contents and language were culturally sensitive and appropriate for the study. YAB members will also be involved in recruiting study participants and in community-led monitoring throughout the study.

### Challenges

Since YAB members in the RISE study are either students, in paid full-time employment or employed as volunteers, most of them encounter challenges in maintaining regular attendance at RISE meetings due to competing life priorities. To mitigate this challenge, meeting scheduling is collaboratively planned between the RISE team and YAB members. Additionally, some YAB members have concerns that their contributions may be undervalued, given their limited experience in formal clinical trial training, however, all RISE YAB meetings and processes explicitly uphold the philosophy that no contribution is meaningless.

The extent of YAB engagement differed across countries because of differences in regulatory policies, environments and institutional processes. For instance, whereas the minimum age of consent to participate in HIV research is 15 years in Nigeria,, Kenya and Malawi, it is 16 years in Zambia.

### Opportunities

Involvement in the RISE YAB has yielded several opportunities for its members, evidenced by their continued active participation and the informal feedback reported. Specifically, RISE offers YAB members personal and professional hands-on development opportunities through meetings, workshops, and networking with the study research team. Currently, YAB members develop meeting agendas and lead meetings, they attend presentations and workshops on implementation science, and as the study progresses, they will spearhead community-led monitoring activities for the study. YABs also serve as platforms for advocacy, enabling members to raise awareness about youth-related concerns, including stigma, mental health, and social justice. With each country’s chair and co-chair participating in the RISE multi-country YAB meetings, they are exposed to further opportunities for networking, cross-learning, and professional growth.

A YAB chair reported ‘*I now feel more confident speaking in front of people, chairing meetings, taking minutes at meeting, and representing my country*.’

Another YAB member reported – ‘*taking part in workshops and discussions about the study has helped board members better understand implementation science concepts, and ethical issues. This makes us feel more legitimate and valuable to the research team*.’

As the RISE study progresses, YAB members will be involved in community-led monitoring, advocacy, and dissemination activities by building on skills already acquired to acquire new research and leadership skills.

### YABs fostering inclusivity

The establishment of YABs in the RISE study demonstrates an inclusive strategy for inclusion of SM-AYA voices in clinical trials by engaging SM-AYA as YAB members to work collaboratively with researchers throughout the duration of the study. RISE engagement in four countries across the SSA region underscores the readiness, responsiveness, interest, and commitment of SM-AYA in being involved in research studies that ultimately benefit their communities. However, sustaining youth engagement requires intentional effort and dedicated resources. These include time, energy, and financial support from clinical trial investigators, and service providers, to optimize YAB members’ confidence in contributing to clinical trials with the recognition that their work must be appropriately compensated [28]. Studies that actively engage youth throughout the research cycle are more likely to produce culturally relevant findings, which in turn enhance individual outcomes, such as professional self-efficacy and agency [20, 28, 29], while also benefiting the broader community, strengthening the clinical trial, and ultimately shaping policy [28, 29].

### Recommendations for establishing future YABs

Our experience in establishing YABs has yielded important recommendations that reflect the lived experiences, needs, and insights of marginalized and under-served populations in African countries. First, periodic training focused on clinical trial methodologies, data analyses, decision-making processes, career growth, and personal development are needed to build the technical capacity of YAB members and improve their ability to meaningfully contribute to clinical trials and enhance their confidence and professional skills. The involvement of YAB members in clinical trials, such as in recruitment, data collection, and demand creation through community outreach, will foster a sense of ownership and contribute to them gaining hands-on experience [30]. Mandoh et al., in their 12-month evaluation study, found that adolescents’ participation in a youth advisory group led to increased built capacity, increased involvement in chronic disease prevention research, and improvements in indicators of adolescents’ leadership and life skills development [20]. Second, to further motivate, legitimize and keep YAB members engaged and productive in clinical trials, other non-monetary incentives, such as public acknowledgments, awards, offers of letters of support, certificates and co-authorship in study manuscripts should be considered to recognize YAB members for their time and contributions [31]. Third, involving YAB members in community-led monitoring activities will enhance equity, service quality, and accessibility by providing their unique perspectives on service delivery challenges leading to more tailored interventions that meet the needs of SM-AYA, promote human rights, and strengthen the overall health program effectiveness [32, 33]. Fourth, creating long-term engagement pathways, such as establishing YAB alumni networks and mentorship programs, will provide ongoing opportunities for former YAB members who step down from their study roles to remain engaged and share their experiences. These networks will serve as platforms for knowledge-sharing, mentorship for new members, and continued community advocacy, thereby ensuring sustained impacts beyond the life of the study. Specifically, before joining the board, new recruited members can be strategically paired with alumni for a couple of weeks for smoother onboarding, sharing experiences, transferring knowledge to boost their confidence, and ease their engagement, integration and transition into their new roles. Fifth, similar to Mandoh et al.’s evaluation of the effects of youth advisory group participation on adolescent’s leadership skills in Australia [20], there is a need to evaluate the effectiveness and impact of YABs on members’ knowledge, leadership skills, and membership retention. Finally, future studies should explore how adolescents and youth engagement through YAB formation translates in professional and personal development.

## Conclusion

As future research studies adopt a similar approach, encouraging a supportive, inclusive and equitable clinical trial environment and addressing existing challenges will be crucial in successfully engaging SM-AYA in relevant health interventions.

## Data Availability

All datasets used and/or analyzed during the current study are available from the corresponding author on request.
